# Bioinspired Cell-Derived Nanovesicles *versus* Exosomes as Drug Delivery Systems: a Cost-Effective Alternative

**DOI:** 10.1038/s41598-017-14725-x

**Published:** 2017-10-30

**Authors:** Wei Jiang Goh, Shui Zou, Wei Yi Ong, Federico Torta, Alvarez Fernandez Alexandra, Raymond M. Schiffelers, Gert Storm, Jiong-Wei Wang, Bertrand Czarny, Giorgia Pastorin

**Affiliations:** 1 NUS Graduate School for Integrative Sciences and Engineering, Centre for Life Sciences (CeLS), Singapore, Singapore; 2 Department of Pharmacy, National University of Singapore, Singapore, Singapore; 3 Department of Anatomy Yong Loo Lin School of Medicine, National University Health System (NUHS), Singapore, Singapore; 4 Singapore Lipidomics Incubator (SLING), Centre for Life Sciences (CeLS), Singapore, Singapore; 5 School of Materials Science & Engineering, Nanyang Technological University, Singapore, Singapore; 6 Clinical Chemistry and Haematology, University Medical Centre Utrecht, Utrecht, Netherlands; 7 Department of Surgery, Yong Loo Lin School of Medicine, National University of Singapore, Singapore, Singapore; 8 Cardiovascular Research Institute (CVRI), National University Heart Centre Singapore (NUHCS) and National University Health System (NUHS), Singapore, Singapore; 9 Department of Pharmaceutics, Utrecht Institute for Pharmaceutical Sciences, Faculty of Science, Utrecht University, Utrecht, Netherlands; 10 NUSNNI-NanoCore, National University of Singapore, Singapore, Singapore

## Abstract

Cell Derived Nanovesicles (CDNs) have been developed from the rapidly expanding field of exosomes, representing a class of bioinspired Drug Delivery Systems (DDS). However, translation to clinical applications is limited by the low yield and multi-step approach in isolating naturally secreted exosomes. Here, we show the first demonstration of a simple and rapid production method of CDNs using spin cups *via* a cell shearing approach, which offers clear advantages in terms of yield and cost-effectiveness over both traditional exosomes isolation, and also existing CDNs fabrication techniques. The CDNs obtained were of a higher protein yield and showed similarities in terms of physical characterization, protein and lipid analysis to both exosomes and CDNs previously reported in the literature. In addition, we investigated the mechanisms of cellular uptake of CDNs *in vitro* and their biodistribution in an *in vivo* mouse tumour model. Colocalization of the CDNs at the tumour site in a cancer mouse model was demonstrated, highlighting the potential for CDNs as anti-cancer strategy. Taken together, the results suggest that CDNs could provide a cost-effective alternative to exosomes as an ideal drug nanocarrier.

## Introduction

Ideally, Drug Delivery Systems (DDS) for therapeutic applications should be capable of site-specific delivery of incorporated therapeutics, escape from recognition and premature degradation by the body’s immune defences and controlled release of cargo molecules upon selective stimuli. A great variety of mostly synthetic DDS have been developed over the past decades to achieve all these desirable properties. Examples of such DDS include nanoscale formulations such as liposomes^1^, carbon nanotubes^2^, gold nanoparticles^3^, solid lipid nanoparticles^4^, micelles^5^, polymeric nanoparticles^6^ and dendrimers^7^. Although showing promising applications, many of these synthetic DDS face issues of poor biocompatibility and lack of intrinsic targeting ability. This is primarily a consequence of the adsorption of plasma proteins onto the nanoparticles’ surface, leading to uptake and activation of immune cells, reduction in circulation time and limited tumour uptake or targeting^7^. Indeed, only a small number of formulations (mainly liposomes and polymeric nanoparticles) have reached FDA approval and found use in clinical setting for cancer therapy^7^. With other DDS, no clinical trials have been reported to date due to sub-optimal tissue distribution profiles and toxicity concerns in pre-clinical investigations^7,8^. Recently, exosomes have gained much attention for their potential use as DDS^9^. These 50–150 nm in size endogenous vesicles function as intermediaries for cell-to-cell communication and are produced by almost all mammalian cells. Indeed, exosomes are cell derived vesicles that are produced through further modification and enrichment at the multi-vesicular body (MVB), resulting in expression of key surface proteins such as Alix, TSG101 and CD9. In addition, exosomes have shown the ability to transport endogenous biological cargo, like small RNAs, mRNAs and proteins across cells^9^. In comparison with current DDS, exosomes are expected to show advantages in terms of biocompatibility and reduced clearance rates in view of their natural origin^10^. Furthermore, they are postulated to show little long-term accumulation in any organ or tissue, with concurrent low systemic toxicity^11^. Besides their biocompatibility, exosomes have shown facilitated cellular uptake as compared to several synthetic DDS^12^. The amount of uptake and route by which the exosomes are internalized are the result of the presence of key proteins on the exosomal surface^13^. In particular, tetraspanins and integrins appear to be important for uptake and intracellular routing^14^.

Furthermore, exosomes have been used to load a variety of small bioactive molecules for drug delivery including paclitaxel^15^, doxorubicin^16^ and curcumin^17^, as well as peptide- or protein-based therapeutics comprising STAT3 inhibitors^18^ and catalase^19^. In addition, loading of exosomes with genetic material such as siRNA has also been reported^20^.

While holding much promise, the use of exosomes as DDS has been hampered by their limited production yield. Large quantities of starting materials consisting of cells and culture medium are necessary in order to achieve the desired amount of exosomes, which require isolation *via* laborious techniques. These nanovesicles are traditionally isolated from culture media in a series of multiple steps *via* differential ultracentrifugation, with further purification through a continuous sucrose gradient^21^. This process is both tedious and time consuming. Other methods of exosome isolation include tangential flow filtration^22^, passage through a size exclusion column^23^ or polymeric precipitation^24^. A common aspect amongst these methods is the relative low yield production of exosomes of approximately 0.5 µg (in terms of protein content) from 10^6^ cells, obtained after a lengthy isolation and purification process^14,21^.

Several strategies have been empirically explored and reported in the literature to circumvent the issue of inefficient production yield. These include prolonging incubation times to lengthen exosome secretion, starting with higher initial cell densities or lowering the pH of culture medium^25^. However, none of these strategies has resulted in a substantially increased production yield or reduced isolation time.

Cell-Derived Nanovesicles (CDNs) offer a promising alternative to the low yields and lengthy purification steps associated with the exosome production and isolation process. CDNs are obtained by subjecting cells to a physical process resulting in vesicles of nano-dimensions. A variety of methods, including passing cells through an extruder^26^, through microchannels^27^ or through custom-made devices fitted inside a centrifuge^28^ have been employed. In the process of CDNs production, the original protein configuration of the parent cells is hypothesized to be conserved^29^. Drawing inspiration from exosomes, CDNs are postulated to mimic several exosomes’ features, resulting in reduced clearance rates and efficient cellular uptake due to the innate targeting ability of the preserved surface proteins^30^. Moreover, using the same number of cells, these methods generate a larger quantity of nanovesicles than traditionally harvested exosomes and over a shorter time period, offering a time saving efficiency of up to 3 days for each production run. Also, CDNs provide additional opportunities of being further amendable to surface functionalization as well as loading of therapeutic cargo, similar to other DDS currently under investigation^14^. While CDNs and exosomes are found to be similar in several aspects, we would expect variations between CDNs and exosomes in terms of loading methods and surface functionalization. These methods reported in the literature for exosomes, such as genetic alteration of precursor cells, metabolic labelling, cell surface modification and hydrophobic insertion, may require different strategies for CDNs as a result of the rearrangement of surface proteins in CDNs and differences in lipid composition^31^.

While strategies for CDNs production offer much higher yields compared to exosomes isolation, current CDNs generation methods have not been fully optimised. These production techniques suffer from relatively low cost-effectiveness, and lengthy processing steps. Due to the need for specialized equipment or high costs, the production of CDNs may still be prohibitive to many laboratories.

**Figure 1:**
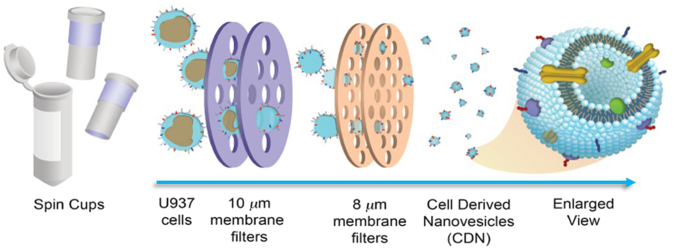
Schematic representation of CDNs production (experimental set-up). Spin cups were fitted with various size membrane filters and U937 cells were forced through the membrane pores to the desired hydrodynamic diameter range using a benchtop centrifuge, adjusting variables of centrifugal force and time to shear cells to obtain CDNs. The dispersion was subsequently passed through a size exclusion column to remove unwanted debris material.

Taking into account all these considerations, here we present a simple and inexpensive method of CDNs production by simply using spin cups fitted with membrane filters of various pore sizes in a benchtop micro-centrifuge, i.e. a common laboratory piece of equipment (Fig. 1). This represents the first demonstration of a simple and rapid production method of CDNs using spin cups *via* a cell shearing approach, which offers clear advantages in terms of yield and cost-effectiveness over both traditional exosomes isolation, and also existing CDNs fabrication techniques.

U937 cells were used as the cell line for production, as these monocyte-derived cells were utilized previously to generate CDNs, and also because the parent cell possesses inherent targeting capabilities that we expect to be passed on to the daughter CDNs^32^. After production and isolation, we compared our CDNs production *vis-à-vis* exosome isolation in terms of yield and processing time, physical characterization, protein configuration and lipidomic profiles. The biological activity of our CDNs was also tested in *in vitro* cellular uptake studies and in *in vivo* biodistribution studies in a mouse model of cancer.

## Experimental Procedures

### Materials

Spin cups were purchased from ThermoScientific, and were supplied with 10μm filters attached while hydrophilic 8μm membrane filters were purchased from Merck Millipore and used as supplied. ThermoScientific micro centrifuge was used in the cell disruption process for the production of cell derived nanovesicles from U937 monocytes. Cyanine 7 and cyanine 5.5 monoester dyes were purchased from Kerafast and used according to the manufacturer’s recommendations. Quantifoil were purchased from Electron Microscopy Sciences.

All methods were performed in accordance with the relevant guidelines and regulations as determined by the National University of Singapore, according to the approval by the Institutional Biosafety Committee (IBC) (Ref number: 2015–00875).

### Cell lines

U937 cells were a kind gift from Assistant Professor Gigi Chiu, National University of Singapore (NUS). CT26 mouse carcinoma cells were kindly provided from Associate Professor Ang Wee Han, NUS. Both cell lines were grown in 10% Fetal Bovine Serum (FBS) supplemented RPMI-1640 cell culture medium. HeLa cells were obtained from American Type Culture Collection (ATCC, Manassas, VA, USA) and grown in 10% FBS supplemented DMEM cell culture medium.

### Cell Derived Nanovesicles (CDNs) and Exosomes production

U937 cells were cultured in 10% FBS supplemented RPMI 1640 medium till 70% confluent, before centrifuging at 500 G for 10 minutes and suspending in Phosphate Buffered Saline (PBS) twice to remove cellular debris and culture medium. Cell suspension of 2 × 10^7^ cell density were placed in a spin cup fitted with a 10 µm membrane filter and centrifuged at 14,000 G for 10 minutes at 4 °C. The flow through was reintroduced to the spin cup and the process repeated. The flow through was then added to another spin cup fitted with two 8 µm membrane filters and the process repeated. The product is then introduced to a Sephadex G50 size exclusion column equilibrated in PBS to further purify the product and fractions corresponding to high protein content and appropriate hydrodynamic diameters were collected.

Exosomes were isolated as described in the literature. Briefly, U937 cells were grown in exosomes depleted medium and centrifuged^21^. The supernatant was collected and underwent differential centrifugation with the final centrifugation done at 100,000 G for 2 hours in a Beckman ultracentrifuge before being resuspended in PBS.

### Physical Characterization of CDNs

Nanovesicles were characterized in terms of hydrodynamic diameter and zeta potential using a Zetasizer Nano (Malvern Instruments). BCA protein assay kit and antibodies were purchased from ThermoScientific and Abcam, respectively and used as supplied.

### Protein marker Analysis of Cell derived Nanovesicles

CDNs and exosomes were added to latex beads and incubated for 2 hours at room temperature, to allow physical adsorption of the samples to the beads. The beads were centrifuged at 3000 G for 10 minutes and suspended in PBS thrice to remove free CDNs or exosomes. The primary antibodies CD9, Alix, and TSG101 were added according to the manufacturer’s recommendations and incubated at 4 °C for 1 hour on a shaker, before being centrifuged and resuspended in fresh PBS. Secondary antibodies conjugated to AlexaFluor 488 or AlexaFluor 568 were added and incubated at 4 °C for 1 hour on a shaker. The beads were washed three times by centrifuging at 3000 G for 10 minutes and suspending in PBS each time. Fluorescence was analysed using the BD LSR Fortessa Flow Cytometry Analyser.

### Lipidomic Analysis

Lipid standards were prepared in butanol: methanol (1:1), of which 200 µl was added to the samples of CDNs, exosomes and U937 cells. The tubes were sonicated for 30 minutes followed by centrifugation at 12000 G at 4 °C. The supernatant was analysed on Agilent 6460 triple quadrupole connected to a UHPLC Agilent 1290 system. A C18 Eclipse plus RRHD 2.1 × 50 mm analytical column with a particle size of 1.8 µm (Agilent Technologies) was used for lipid analysis. The solvents used were 40% acetonitrile in 60% water with 10 mM ammonium formate (Mobile phase A) and 10% acetonitrile in 90% isopropanol with 10 mM ammonium formate (Mobile Phase B). The peak areas of the lipids were integrated using Agilent Mass Hunter Quantitative Analysis Software. Peak areas were normalized to the internal standards.

### Cryo-TEM imaging

Lacey Formvar/Carbon, 300 mesh Copper grids (TED PELLA INC., USA) were loaded with 30 μL of sample. After a blotting time of 2 seconds, TEM grids were subjected to plunge-freezing into liquid ethane using Gatan CP3 Cryo-plunger 3 system (Gatan, Pleasanton, CA). Cryo-grids were then transferred at liquid nitrogen temperature and imaged with a Carl Zeiss TEM, LIBRA® 120 PLUS, provided with advanced Koehler illumination system and operated with accelerating voltage of 120KeV^33^. Images were recorded at a magnification between 80,000–10,000x, diameter size of the condenser aperture 500 µm and the brightness used was 60 µradians with camera resolution, 0.34 nm from point to point.

In order to visualize individual vesicles, CDNs and exosomes were fixed using 2.5% glutaraldehyde and placed onto a Quantifoil with 2 seconds blotting time. The samples were then plunged into liquid ethane using Gatan CP3 Cryo-Plunger 3 and viewed using JEOL JEM-2200FS.

### Stability Studies

The hydrodynamic diameters and stability of CDNs were investigated in relation to the temperature stored and the buffer system used over 4 weeks (Figure S1). CDNs were either stored at 4 °C or 25 °C for the temperature study. PBS or normal saline were used for buffer systems, as these buffer systems are commonly found in the clinical setting. Triplicates were performed.

### Labelling of CDNs using Cyannine7 and Cyanine 5.5 monoester dyes

Cell Derived Nanovesicles were labelled with cyanine 5.5 *N*-hydroxysuccinimide (NHS) monoester by adding the dye as per manufacturer instructions at pH 8 and incubating for 4 hours on ice. The labelled CDNs were then returned to pH 7 and free dye was removed by overnight dialysis in PBS, before being introduced to a Sephadex G50 size exclusion column equilibrated with PBS. Fractions of 500 µl were collected and analysed for protein content using a standard BCA protein assay kit and for fluorescence corresponding to excitation/emission of 673/700 nm (Figure S2). CDNs were labelled with cyanine 7 NHS (N-Hydroxysuccinimide) monoester in the same manner.

### Cellular uptake studies of CDN_cy5.5_

CDN_cy5.5_ as described previously was added to HeLa cells at 37 °C, 5% CO_2_. The samples were incubated for 6 hours before adding Hoechst 33342 dye and CellMask Orange for nuclei visualization and membrane staining respectively. Samples were washed at least thrice with sterile PBS to remove free CDNs before fixing with 4% paraformaldehyde and were imaged using a confocal microscope (FLUOVIEW FV10i Olympus).

To elucidate if the cellular uptake mechanism of cell derived nanovesicles were *via* an energy dependent process, CDN_cy5.5_ were added to HeLa cells and incubated for 4 hours at 37 °C, 20 °C, 4 °C. To further investigate the route of cellular uptake, caveolae inhibitor genistein (200 µM) and clathrin inhibitor chlorpromazine (10 µg/ml) were added respectively to HeLa cells, and incubated for at least 30 minutes before addition of samples under the same conditions. Hoechst 33342 dye was added for live cell visualization. The HeLa cells were subsequently washed three times with sterile PBS, trypsinized and assayed using the BD LSR Fortessa Flow Cytometry Analyser.

### *In Vivo* Experiments

All animals models used were approved by the Institutional Animal Care and Use Committee (IACUC) at National University of Singapore (NUS). CT26 mouse colon adenocarcinoma cells were grown till 70% confluent and injected into the subcutaneous layer at the back of white BALB/c mice at 5 weeks old at a cell concentration of 1 × 10^5^ cells/50 µl, suspended in sterile PBS. The tumour was allowed to develop into an appropriate size for 1 week before injecting the samples intravenously into the tail vein of the mice. Three mice groups consisting of 5 mice each were used for CDN_cy7_, Exo_cy7_ and free Cy7 dye samples respectively. Cyanine 7 NHS monoester were labelled onto CDNs and exosomes in the same manner as Cyanine 5.5 NHS monoester. Cyanine 7 was chosen for *in vivo* labelling to avoid any interference from eventual tissues’ auto-fluorescence. The samples were injected intravenously (40 µg/100 µl) into the tail vein and fluorescence visualized after 24 hours using *In Vivo* Imaging Systems (IVIS) spectrum (Caliper Life Sciences). The mice were sacrificed after 24 hours to quantify the fluorescence intensity of the organs using IVIS Living Image Version 4.4.

### Statistical Analysis

All statistical analysis were performed using IBM SPSS Version 21. Lipidomic analysis and radiant efficiencies of *in vivo* experiments were compared using one way ANOVA statistical test, using Bonferroni post hoc tests. P values less than 0.05 were considered to be significant.

## Results and Discussion

### Production of CDNs

For CDNs production, U937 cells were added to spin columns fitted with various size membrane filters and sheared to the desirable size using the centrifugal force provided by a micro-centrifuge (Fig. 1). CDNs were produced by sequential centrifugation of the cell suspension, using 10 µm and then 8 µm membrane filters. The cells underwent a size reduction through shear forces and the CDNs produced were further purified *via* size exclusion chromatography column to obtain the fractions with dimensions, below 300 nm (namely 50–200 nm), which is a desirable size to take advantage of the Enhanced Permeability and Retention (EPR) effect without premature clearance from the body^11^.

### CDNs characterization and comparison

**Figure 2:**
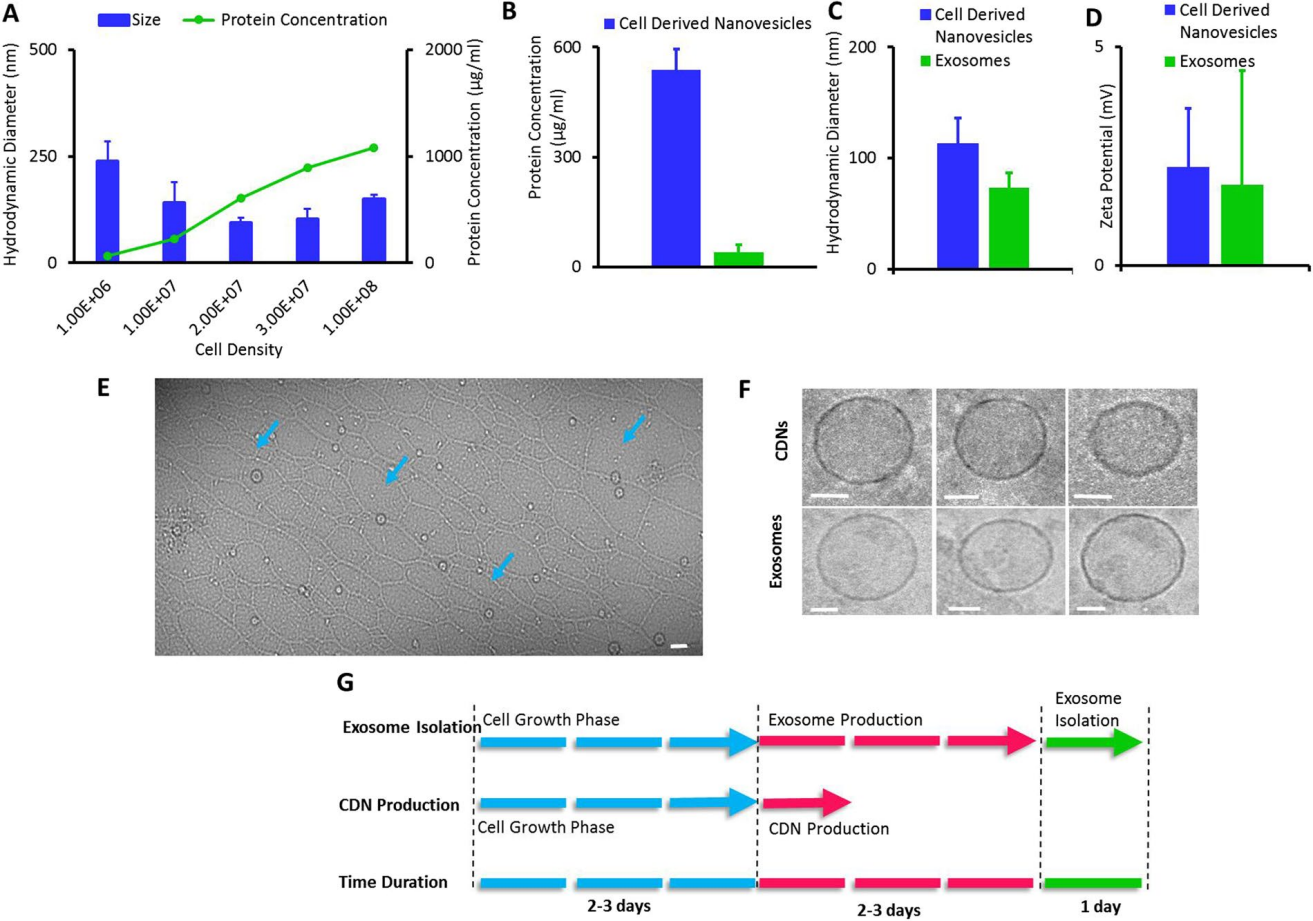
Physical Characterization of Cell Derived Nanovesicles (CDNs) obtained from 2 × 10^7^ U937 cells based on parameters of (**A**) CDNs hydrodynamic diameter, cell density, protein concentration, and (**B**) Comparison of protein yield of CDNs and exosomes. (**C**) Hydrodynamic diameter comparison of CDNs and exosomes. (**D**) Zeta potential comparison of CDNs and exosomes. (**E**) Cryo-TEM images of CDNs (blue arrows). Scale bar indicates 100 nm. (**F**) Comparison Cryo-TEM images of CDNs (top row) and exosomes (bottom row). Scale bars depict 20 nm. (**G**) Time saving efficiencies with CDNs production compared to exosome isolation.

Interestingly, tuneable control of CDNs’ hydrodynamic diameter could be achieved by adjusting the initial cell density (Fig. 2A). The general observed trend was that a smaller mean hydrodynamic diameter of CDNs was obtained when a higher cell density of U937 cells was used. Dynamic light scattering (DLS) measurements indicated a hydrodynamic diameter reduction to less than 200 nm when at least 1 × 10^7^ cells were used. A minimal mean CDNs hydrodynamic diameter of 110 nm was obtained when 2 × 10^7^ cells were used, comparable to other CDNs production methods that created CDNs of about 130 nm in hydrodynamic diameter^26^. No CDNs were detected when less than 1 × 10^6^ cells were used, indicating a minimum cell density required for nanovesicles formation. Increasing cell density from 2 × 10^7^ cells to 1 × 10^8^ cells did not reduce the hydrodynamic diameter of the CDNs any further. While the mechanism of the lower cell density needed for production is still under investigation, we postulate that a relationship exists between the number of cells and size of the membrane filter. Given that only a certain number of cells may pass through the membrane filters at any one time, increasing the number of cells would result in greater cell-to-cell attrition. This hypothesis may also explain why the hydrodynamic radius is affected by cell density: an increase in cell density results in greater cell-to-cell attrition and therefore reduced hydrodynamic radius. Other permutations of the production process, such as membrane pore size, number of membrane filters, sequence of membrane filters and centrifugation force, did not produce CDNs of appropriate hydrodynamic diameters between 50 and 200 nm (data not shown).

Protein concentration of CDNs produced at varying initial U937 cell densities, from 1 × 10^6^ to 1 × 10^8^ cells, was determined using a standard protein assay (Fig. 2A). An increase in cell density corresponded to a rise in protein concentration, suggesting that scalability in CDNs production can be achieved by simply increasing the cell density.

We also compared CDNs from our production method with exosomes isolated using the ultracentrifugation process. In our method of CDNs production, the yield in protein concentration was found to be about 15 fold higher than conventional exosome isolation (540 µg/ml for CDNs production and 40 µg/ml for exosome isolation, with both samples produced from 2 × 10^7^ cells/ml), further demostrating the cost-effectiveness of this CDNs production method (Fig. 2B). Moreover, our approach for CDNs had a higher total protein yield (540 µg/ml) compared with other CDNs reports in the literature, that reached 406 µg/ml of total proteins, derived from the same cell density of 2 × 10^7^ cells/ml^26^.

In addition, hydrodynamic diameter (Fig. 2C) and zeta potential (Fig. 2D) of CDNs were also compared with exosomes and found to be similar, also in terms of morphological evaluation under Cryo-TEM (Fig. 2E and F).

When we compared the time needed to obtain the final product of exosomes or CDNs (at the same starting cell density) (Fig. 2G), exosomes production and isolation required up to 4 days, whereas CDNs production took as little as less than a day. This, in turn, offers time saving of 3 days per production run. This reduction in processing time is mainly due to the need for exosomes’ secretion from cells in exosome-depleted culture medium before finally isolating the desired exosomes. This step is absent in CDNs production, where CDNs could be produced on demand once the desired cell density is obtained.

### Protein Marker Analysis

**Figure 3:**
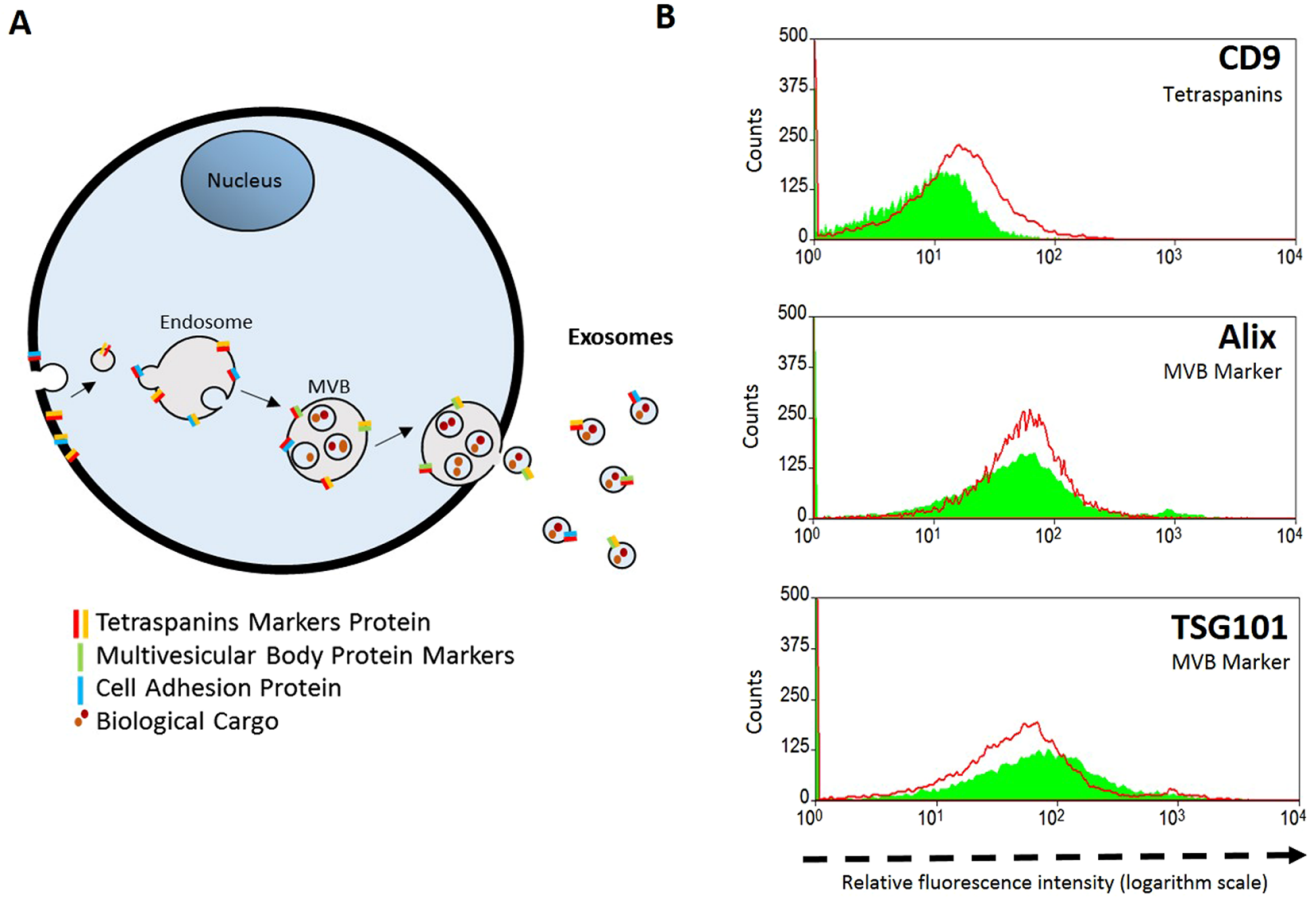
Comparison of Cell Derived Nanovesicles (CDNs) with Exosomes. (**A**) Biogenesis of exosomes and associated protein markers. (**B**) Flow cytometry of three key protein markers’ (Tetraspannins: CD9, Multivesicular Body Markers (MVB): Alix and TSG101) distribution in CDNs and isolated exosomes. CDNs and exosomes are depicted in green and red, respectively.

Exosomes are formed from the inward invagination of endosomes and are further modified and enriched at the MVB, retaining protein markers of the cell membrane and also those acquired at the MVB level (Fig. 3A)^13,34^. Two groups of protein markers, namely tetraspannins (CD9) and multivesicular body protein markers (ALIX and TSG101) are considered canonical exosome markers. We compared these proteins on exosomes and CDNs to investigate whether the composition of CDNs was similar to isolated exosomes (Fig. 3B)^35^. The results confirmed that our CDNs mimicked natural exosomes. Relevant protein markers that are enriched at the MVB during exosome biogensis are present on the CDN surface (Fig. 3B), whereby the close similarities in key protein configurations in both exosomes and CDNs is suggestive that the most relevant functional features of exosomes for drug delivery, namely low clearance rates and specific cellular uptake, are preserved in our CDNs. Future studies on the elimination rate of exosomes *versus* our CDNs will be needed to confirm this hypothesis.

### Lipidomic profiles

**Figure 4:**
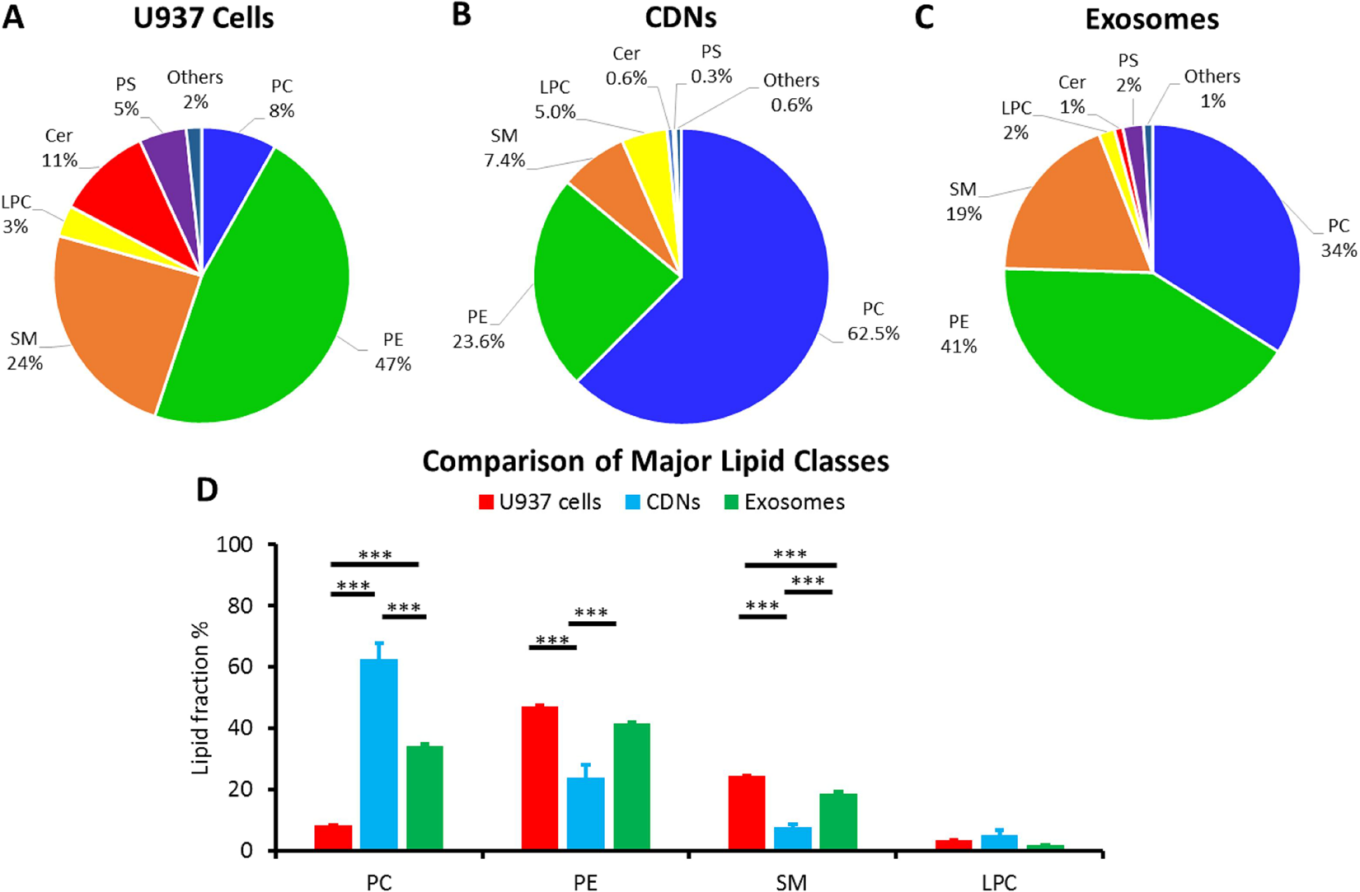
Comparison of total lipid content of (**A**) U937 cells, (**B**) CDNs and (**C**) exosomes derived from 2 × 10^7^ U937 cells. (**D**) Lipid comparison of 4 major classes in U937 cells, CDNs and exosomes. Lipids: Sphingomyelin (SM), Phosphatidylserine (PS), Ceramide (Cer), Phosphatidylcholine (PC), Phosphatidylethanolamine (PE), and Lysophosphatdiylcholine (LPC). ***Indicates P < 0.001.

Beside protein analysis, we were also interested at the comparison of the lipidic consituents of CDNs with exosomes and the parent cells (Fig. 4A–D). The major lipid consituents of exosomes were found to be retained by CDNs in the production process and with the same abundance: phosphatidylcholine, phosphatidylethanolamine, sphingomyelin and lysophosphatidylcholine were present in both exosomes and CDNs. This, in turn, is broadly different from the lipidomic profile of U937 cells, where the main consituents in order of abundance are phosphatidylethanolamine, sphingomyelin, ceramide and phosphatidylcholine, showing that CDNs are generally more similar to exosomes than the original U937 cells in terms of composition of major consituents. More importantly, the similarities in major lipidomic consituents imply that characteristics pertaining to lipid type such as fluidity, deformability, stability and potential leakiness are largely similar, suggesting the potential ability of CDNs to act as “exosome mimetics”^29^.

However, we noted that the ratio of phosphatidylcholine (PC) to the other lipids in CDNs was much higher than that in exosomes and in the parent U937 cells. This difference in lipid ratios may allude to the different properties of each vesicle type (Fig. 4D). Interestingly, the high phosphatidylcholine profile of CDNs shares some resemblance to erythrocytes, where 65% to 75% of phosphatidylcholine resides in the exterior^36^. Furthermore, a lower percentage of phosphatidylserine (PS) (0.3%) was observed in CDNs as compared to exosomes (2%) and U937 cells (5%). Overexpression of phosphatidylserine is postulated to increase clearance by the Mononuclear Phagocyte System (MPS), thereby reducing circulation time *in vivo* ^36^. Taken together, the reduction in phosphatidylserine and the similarity to erythrocytes may further aid in improving cellular uptake and *in vivo* circulation time.

### Mechanisms of cellular uptake and fate *in vitro*

**Figure 5:**
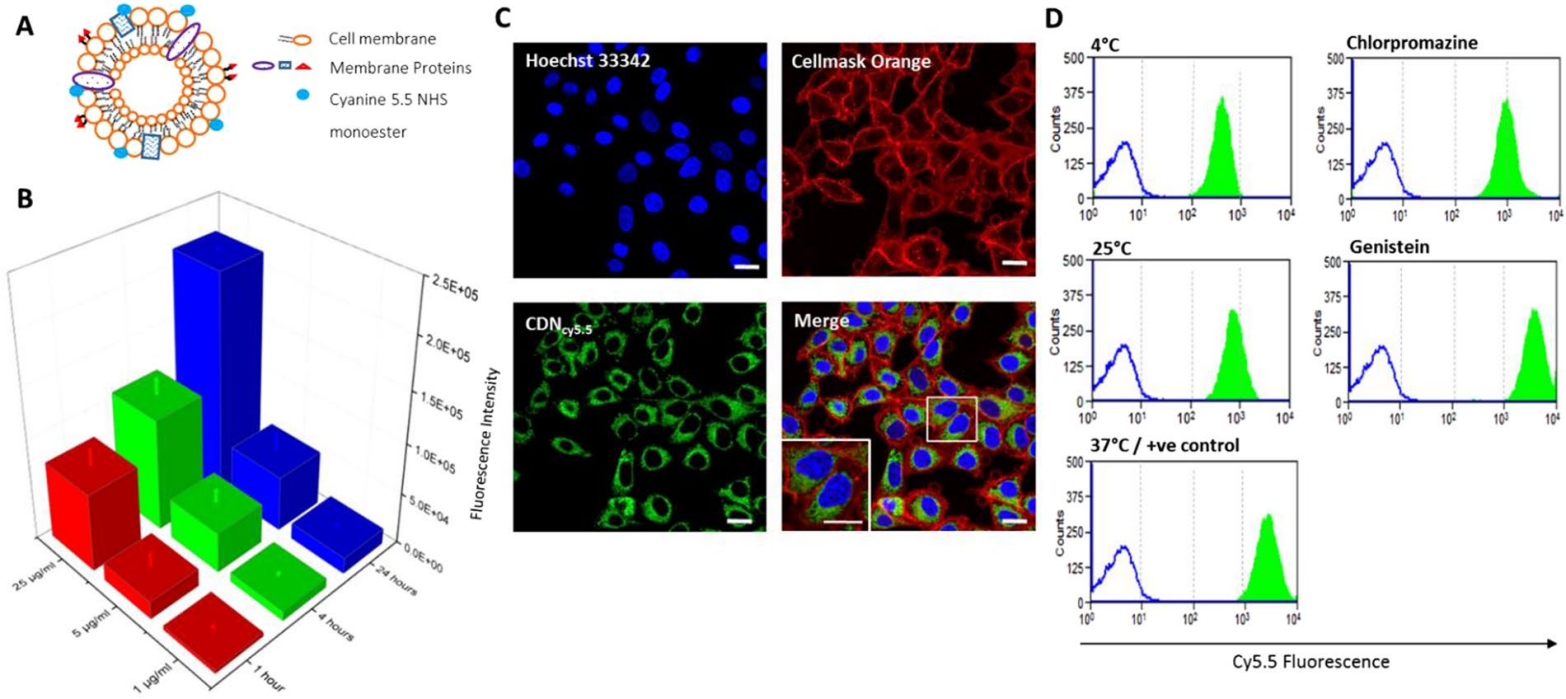
(**A**) Labelling of CDNs’ surface by Cyanine 5.5 monoester dye to obtain cyanine 5.5-labelled CDNs (CDN_cy5.5_). (**B**) Cellular uptake of CDN_cy5.5_ by HeLa cells after incubation for 1 hour, 4 hours and 24 hours at protein concentrations of 1 µg/ml, 5 µg/ml and 25 µg/ml. The cells were washed twice with sterile PBS before being trypsinized and assayed *via* FACS. (**C**) Confocal microscopy images of CDN_cy5.5_ 4 hours post incubation with HeLa cells. Nuclei and plasma membrane of Hela cells were stained with Hoechst 33342 and CellMask Orange, respectively. Scale bars indicate 20 µm. (**D**) Cellular uptake of CDN_cy5.5_ at 3 temperatures, 4 °C, 25 °C and 37 °C, as well as in the presence of chlorpromazine (25 µM) and genistein (200 µM) 4 hours post incubation, to elucidate the cellular uptake mechanism of CDNs. Blue outline indicates unstained HeLa cells, green areas indicate HeLa cells upon treatment.

To investigate the cellular uptake of CDNs *in vitro*, CDNs were labelled with cyanine 5.5 NHS monoester (Fig. 5A). Increasing concentrations (1 µg/ml, 5 µg/ml and 25 µg/ml based on protein content) of CDN_cy5.5_ were added to HeLa cells, which were incubated for 1, 4 and 24 hours (Fig. 5B). CDN_cy5.5_ cellular uptake was found to be both time- and dose-dependent. The CDN_cy5.5_ perinuclear internalization as CDN_cy5.5_ (green) was positioned between the nucleus (blue) and plasma membrane (red) (Fig. 5C). Free dye without CDNs did not label cells.

Additionally, we explored the cellular uptake mechanism of CDN_cy5.5_ by incubating HeLa cells at various temperatures of 4 °C, 25 °C and 37 °C (Fig. 5D). Lowering the incubation temperature reduced the uptake of CDN_cy5.5_ into the cells by energy-driven processes such as endocytosis^37^. Incubating HeLa cells with chlorpromazine and genistein inhibited the clathrin and caveolae-mediated endocytosis pathways, respectively. The results indicated that the uptake of CDN_cy5.5_ was more affected in the case of pretreatment with chlorpromazine than with genistein^38^. Since the hydrodynamic diameter of CDNs is close to 100 nm, this corresponds closely to the hydrodynamic diameters of vesicles that are involved in clathrin-mediated endocytosis, whereas vesicles involved in caveolae-mediated endocytosis tend to be smaller (between 60 to 80 nm), suggesting that the clathrin-mediated endocytosis pathway may be the main contributor to the cellular uptake mechanism of CDNs^39^. The results also corroborate on the cellular uptake mechanisms of exosomes, which occur through clarthin-dependent endocytosis, as well as other mechansims of macropinopcytosis, phagocytosis and lipid-raft mediated internalization^40^. This, in turn, confirms once again the close similarities in terms of surface configuration between exosomes and CDNs. Indeed, data in the literature suggest that, while endocytosis is the primary mode of celullar uptake, exosomes populations are often heterogenous and may be taken up by cells *via* more than one mechanism, and the same would be expected of CDNs^40^.

Lastly, addition of unloaded CDNs showed little toxicity to HeLa cells across a range of concentrations, suggesting a drug delivery approach associated with low intrinsic cytotoxicity, similar to that of exosomes (Figure S3).

### *In vivo* biodistribution of CDNs in a mouse tumor model

**Figure 6:**
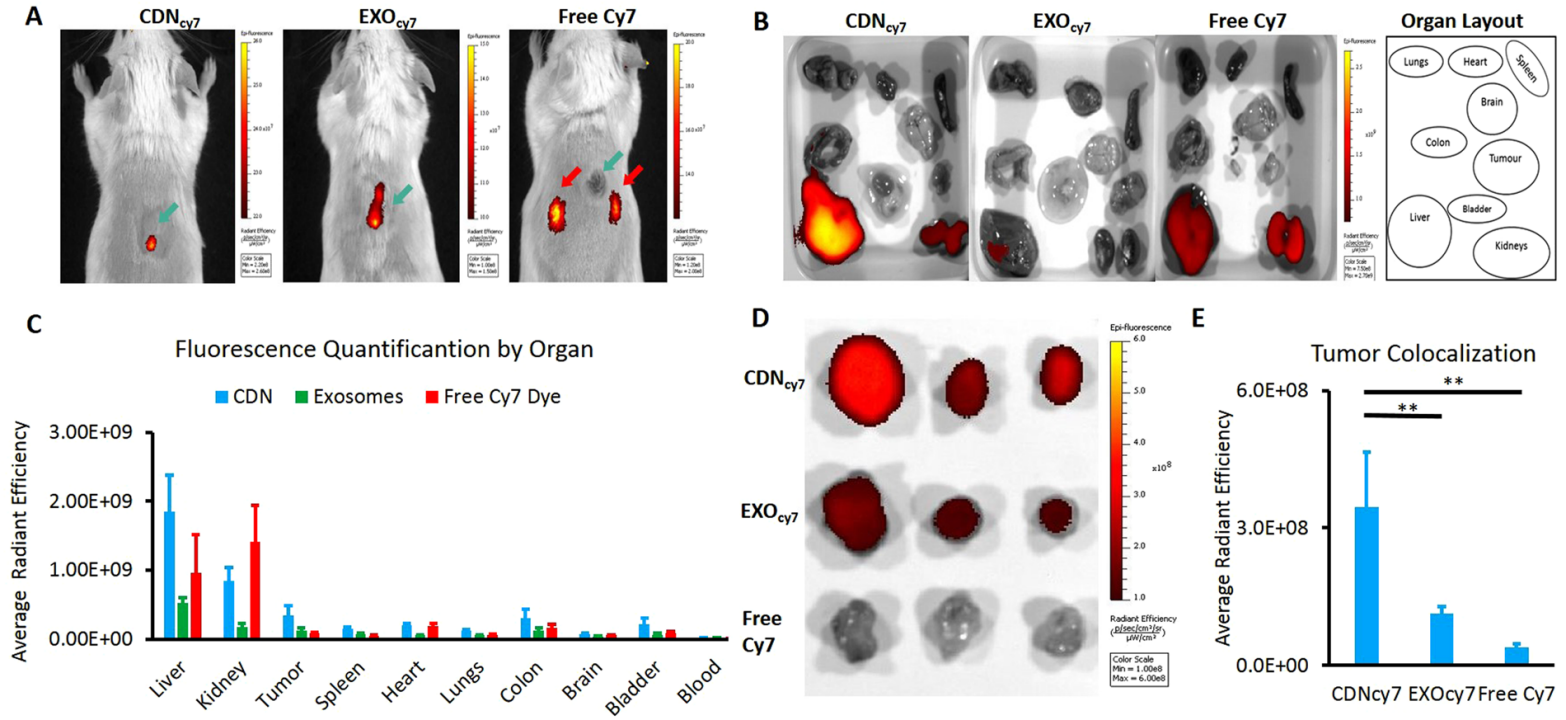
IVIS imaging of biodistribution of Cyanine 7-labelled CDNs, exosomes and free Cyanine 7 dye in mice tumour model. Tumour sites are denoted by green arrows. (**A**) Cyanine 7-labelled CDNs (CDN_cy7_) and exosomes (Exo_cy7_) accumulated at the tumour site 24 hours after intravenous (IV) injection. No accumulation was observed in the tumour for mice injected with free Cyanine 7 dye, where accumulation at the kidneys was observed instead, as denoted by red arrows. (**B**) Accumulation of CDN_cy7,_ Exo_cy7_ and free dye in various excised organs 24 hours after injection. (**C**) Quantification of Cy7 radiance efficiency in various organs 24 hours after injection. (**D**) Representative IVIS images of excised tumours. (**E**) Quantification of Cy7 radiance efficiency in excised tumours. **Indicates P < 0.01.

Finally, we also compared CDNs to exosomes in terms of *in vivo* tissue distribution in a mouse tumour model. CDNs and exosomes were labelled with Cyanine 7-NHS monoester dye normalized to protein concentration and injected intravenously into BALB/c mice engrafted with syngeneic CT26 colon adenocarcinoma and imaged using *In Vivo* Imaging Systems (IVIS) Living Image 24 hours post injection. Cyanine 7 (Cy7) fluorescence was observed in the tumours (indicated by green arrows) of mice injected with Cy7-labelled CDNs (CDN_cy7_) and with Cy7-exosomes (Exo_cy7_) but not with free Cy7 (Fig. 6A). Accumulation of free Cy7-NHS dye was observed in the kidneys (indicated by red arrows) with no fluorescence observed at the tumour site, suggesting that rapid elimination of the free dye occurred *via* the kidneys and that the circulation time of the CDNs and exosomes were at least 24 hours (Fig. 6A). Negative controls of fluorescence labelled CDNs injected in tumour free mice and PBS injected into tumour mice model can be found in Figures S4 and Figures S5 respectively.

The quantification of Cy7 in the organs showed the liver as the main accumulation site for CDNs and exosomes (Fig. 6B and C). This underlines that the liver plays a dominant role in the clearance and eventual elimination of both CDNs and exosomes. The free dye exhibited a high accumulation in the kidneys as compared to CDNs and exosomes, indicating that elimination of free dye is primarily *via* the renal excretion pathway. Fluorescence was also observed in other organs, though at much lower intensities. Interestingly, higher fluorescence levels were empirically observed in the colon faecal material in mice injected with CDN_cy7_ and Exo_cy7_ as compared to free Cy7 dye, suggesting that at least some of the CDN_cy7_ and Exo_cy7_ were trapped possibly by the Kupffer cells in the liver and eliminated through the faeces in a manner similar to that occurring in the case of liposomes^41^.

Moreover, strong fluorescence was observed in the tumour for mice injected with highest intensities in CDN_cy7_, followed by Exo_cy7_, as compared to the free Cy 7 dye, further reinforcing the notion of the accumulation of CDNs at the tumour site *via* EPR effect, similar to that of exosomes (Fig. 6D and E). While the fluorescence levels found in tumours injected with CDN_cy7_ were significantly higher (P < 0.01) than those of Exo_cy7_ and free Cy7 dye, we are unable to attribute this observation to the higher targeting effect of CDNs vis-à-vis exosomes. Instead, it would seem more likely that a greater concentration of fluorescent tags are found on CDNs than exosomes. This may be due to the presence of more protein (to which the dye was attached covalently) at the surface of CDNs than exosomes; therefore, a greater fluorescence intensity was observed at the tumour site in the case of CDN samples. Nonetheless, the presence of strong fluorescent signal in the tumours for both CDNs and exosomes indicate a targeting effect towards the tumour site. This targeting effect may be attributed to the small size of both CDNs and exosomes: the hydrodynamic diameter of the CDNs falls below 200 nm, allowing them to exploit the EPR effect, thus entering the leaky vasculature surrounding the tumour site and accumulating in a fashion similar to exosomes.

The accumulation of CDNs at the tumour site could aid in the reduction of off targeting effects, thereby mitigating adverse reactions should a chemotherapeutic agent be loaded. Furthermore, in a previous study we demonstrated our CDNs’ preferential targeting to HeLa cancer cells, even when co-cultured with HEK293 cells of non-cancereous origin^42^. Therefore, CDNs represent promising nanocarriers for delivery of chemotherapeutics and hold great potential in the still enigmatic treatment of cancer^43^.

## Conclusions

We demonstrated an improved and cost-effective production method of CDNs and verified that the CDNs retained the membrane protein configuration of the original cells. More importantly, CDNs were produced in a higher yield and more cost-effective manner than conventional exosomes isolation methods and also current CDNs production methods. Our approach could also be potentially scaled up to larger size tubes to be used in common laboratory centrifuges, with each batch of CDNs confined within a single container, thus limiting cross contamination and allowing for large scale production. Characterization studies and comparison between exosomes and CDNs in terms of physical attributes, key protein markers, lipidomic profiles as well as *in vitro* and *in vivo* behaviour were investigated, and found to be highly similar in almost all aspects. Here, we envision a strategy where CDNs hold promising potential as a viable cost-effective alternative to exosomes for applications in drug delivery.

## Electronic supplementary material

Supplementary Information
