# Supplementary material for: Bioinspired Cell-Derived Nanovesicles versus Exosomes as Drug Delivery Systems: a Cost-Effective Alternative
Source: Sci Rep. 2017 Oct 30;7:14322. doi: 10.1038/s41598-017-14725-x (PMC5662560; doi:10.1038/s41598-017-14725-x)
Supplement: Supplementary file 1 — Supplementary Information [file 41598_2017_14725_MOESM1_ESM.pdf]

Bioinspired Cell-Derived Nanovesicles *versus* Exosomes as Drug Delivery Systems: a Cost-Effective Alternative

By Wei Jiang Goh, Zou Shui, Wei Yi Ong, Federico Torta, Alvarez Fernandez Alexandra, Raymond M. Schiffelers, Gert Storm, Jiong-Wei Wang, Bertrand Czarny* and Giorgia Pastorin*

Supplementary Information

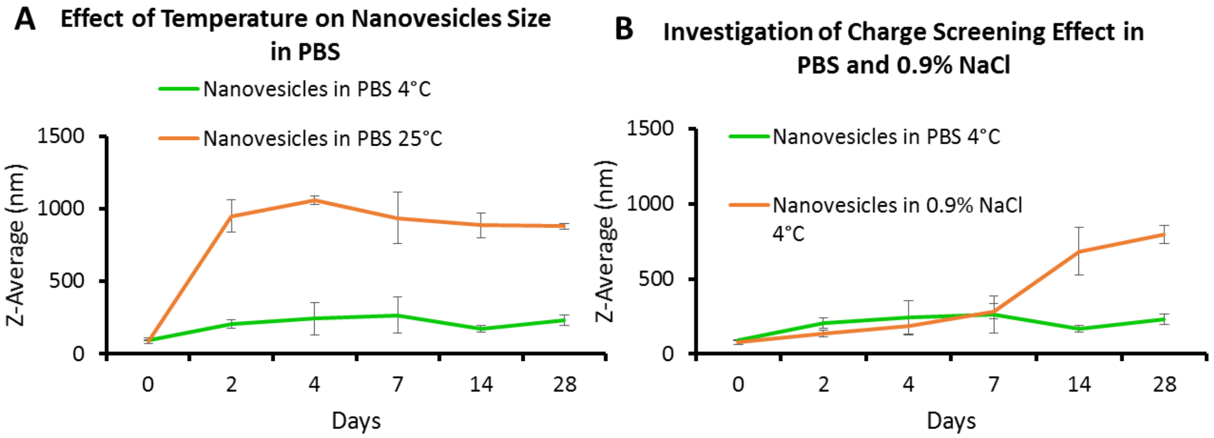

Figure S1. CDNs stability studies measuring increasing in size and aggregation of CDNs over 4 weeks relating to (a) Temperature and (b) different buffer systems using PBS and Normal Saline at 4°C.

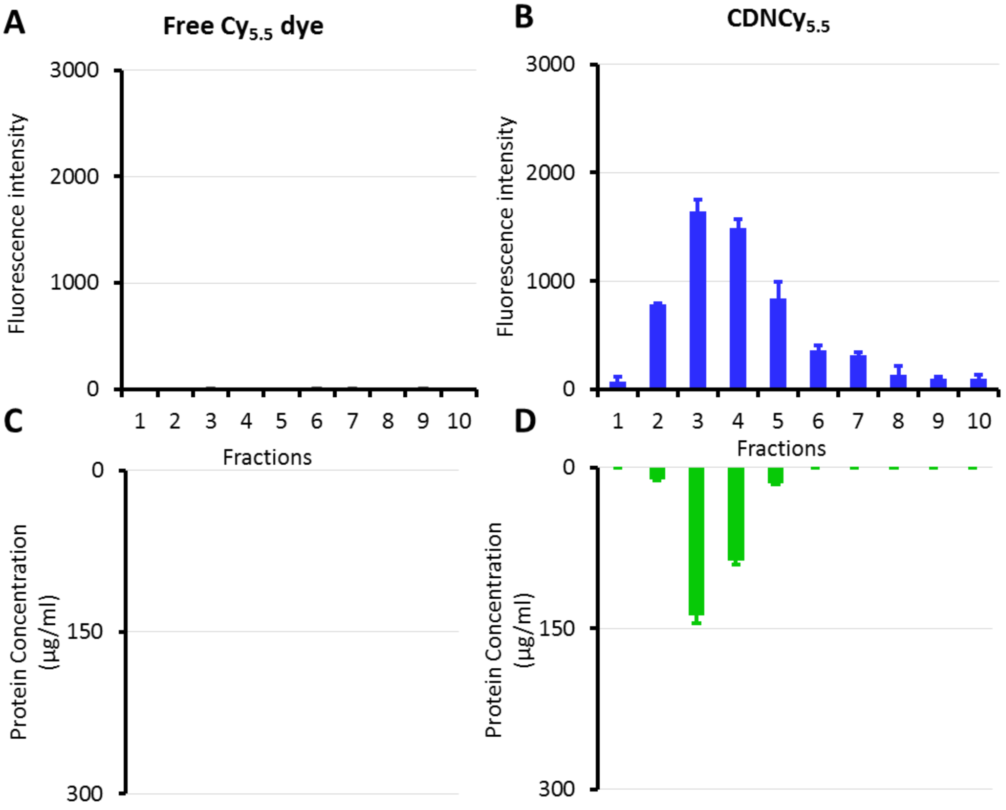

Figure S2. Fractions collected from Size Exclusion Chromatography were assayed for fluorescence intensity of the Cyanine 5.5 dye, using (A) free dye and (B) $CDN_{cy5.5}$. The corresponding fractions were also assayed for protein concentrations of (C) free dye and (D) $CDN_{cy5.5}$. Free dye fractions were diffused and did not exhibit substantial fluorescence nor protein concentrations, whereas $CDNcy_{5.5}$ showed a peak in fluorescence values at fractions 3 and 4, together with corresponding peak protein values at the same fractions.

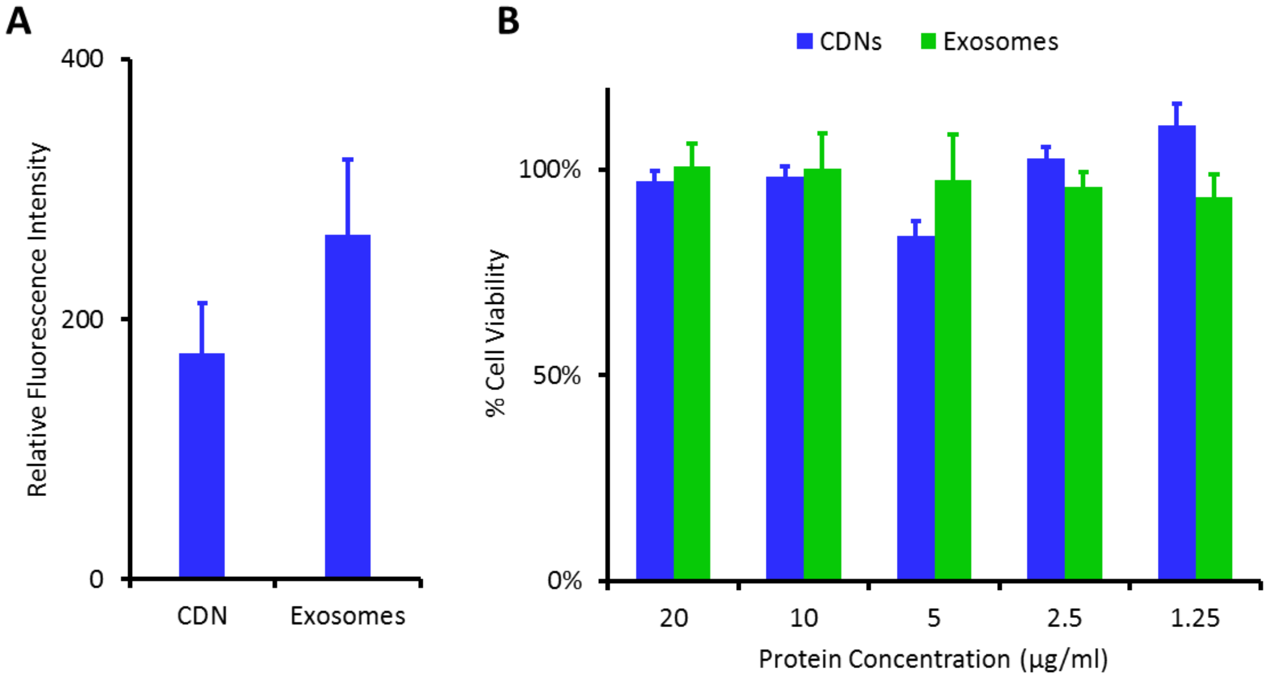

Figure S3. (A) Analysis of relative fluorescence intensity of CDNs and exosomes, with normalized protein concentrations, stained with Hoechst 33342 dye, as an indication of amount of DNA within the vesicles. Analysis was done by adsorbing CDNs and exosomes onto beads before staining with Hoechst 33342 and analysis *via* flow cytometry. (B) Effect of empty CDNs and exosomes on HeLa cell viability using standard MTT assay. HeLa cells were incubated with normalized protein concentrations of CDNs and exosomes for 48 hours.

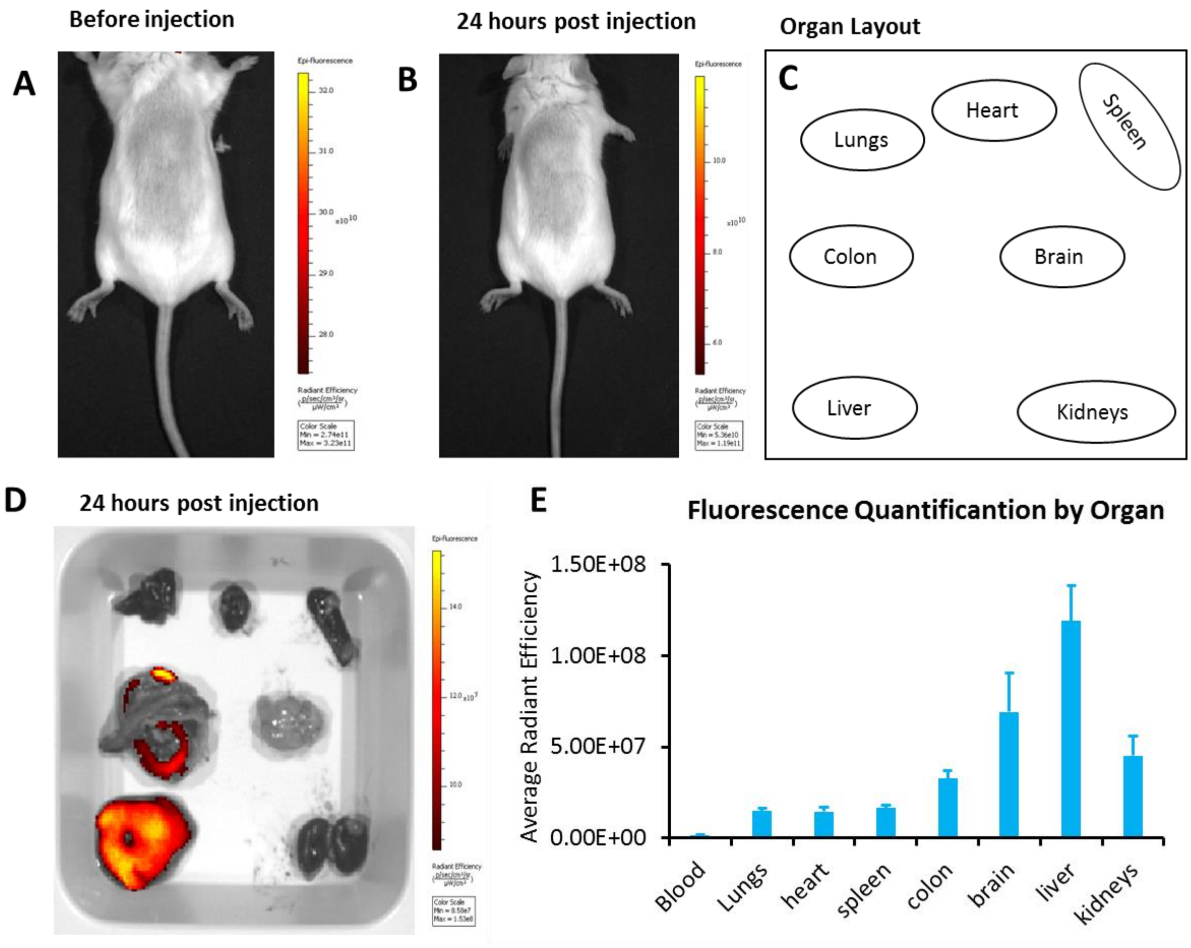

Figure S4. (A) IVIS image of representative mice without tumour before tail vein IV injection with Cy5.5 labelled CDNs. (B) IVIS image of mice without tumour post 24 hours after injection with Cy5.5 labelled CDNs. (C) Organ layout schematic. (D) IVIS imaging of excised organs 24 hours post injection. (E) Fluorescence quantification of excised organs 24 hours post injection.

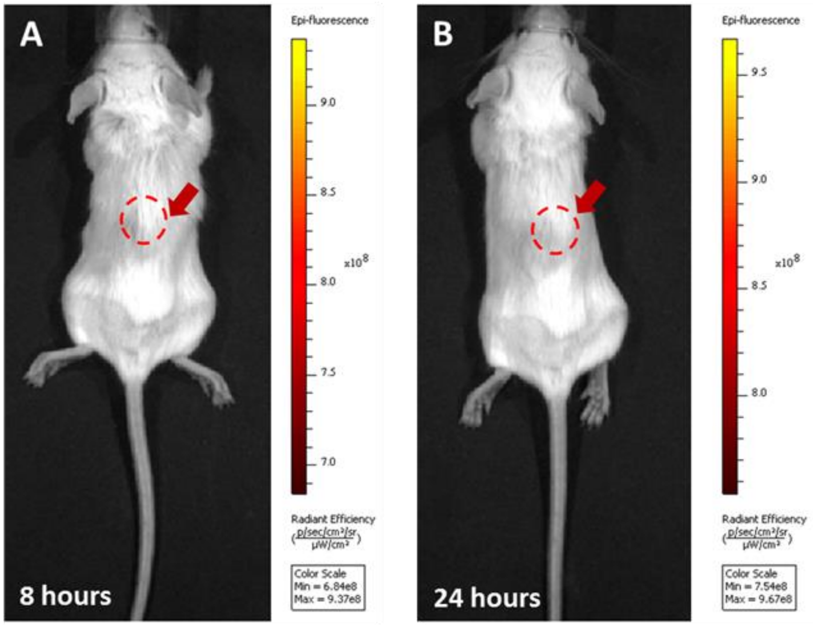

Figure S5. Mice tumour model after IV tail vein injection of PBS and imaged for cyanine 7 fluorescence at (A) 8 hours and (B) 24 hours. Tumour positions are marked by red arrows.
